# Electronic Devices and Digital Therapeutics in Neuromuscular Diseases: Narrative Review on Current Applications in Patients' Management and Pharmacovigilance

**DOI:** 10.1002/hcs2.70108

**Published:** 2026-10-08

**Authors:** Ilaria Saltarella, Concetta Altamura, Paola Laghetti, Giuseppina Settanni, Nicola Savino, Angelo Galiano, Bodvaël Fraysse, Angelo Vacca, Jean‐François Desaphy

**Affiliations:** ^1^ Section of Pharmacology, Department of Precision and Regenerative Medicine and Ionian Area School of Medicine, University of Bari Aldo Moro Bari Italy; ^2^ Interdepartmental Center for Research in Telemedicine University of Bari Aldo Moro Bari Italy; ^3^ R&D Department Dyrecta Lab, Research Institute Conversano Italy; ^4^ Nantes Université, CHU de Nantes, INSERM UMR 1089, Translational Gene Therapy Laboratory (TaRGeT) Nantes France; ^5^ Section of Internal Medicine, Department of Precision and Regenerative Medicine and Ionian Area School of Medicine, University of Bari Aldo Moro Bari Italy

**Keywords:** digital therapeutic, digital therapy, electronic device, neuromuscular disease, pharmacovigilance

## Abstract

Digital health technologies have revolutionized the management of neuromuscular diseases (NMDs), offering novel opportunities for disease monitoring, rehabilitation, telemedicine, pharmacological research, and pharmacovigilance. This narrative review aims to provide an integrated overview of the current applications of digital health technologies in NMDs, highlighting their role in diagnosis, disease monitoring, rehabilitation, telemedicine, pharmacological research, and pharmacovigilance. Relevant articles were identified by interrogating PubMed using combinations of keywords including “neuromuscular diseases,” “digital health,” “digital therapeutics,” “telemedicine,” “wearable devices,” “remote monitoring,” “telerehabilitation,” and “pharmacovigilance.” This review describes how wearable devices, digital biomarkers, telemedicine platforms, and digital therapeutics can facilitate continuous and real‐time monitoring of clinically relevant parameters, including muscle strength, mobility, physical activity, and disease progression. In addition, digital technologies support remote patient management, rehabilitation programs, early detection of clinical changes, and the development of personalized therapeutic interventions. Emerging evidence also suggests that digital health tools may improve the design and conduct of clinical trials, enhance the collection and analysis of real‐world data and patient‐reported outcomes, and contribute to more effective pharmacovigilance strategies. Despite challenges on data privacy, digital literacy, integration into healthcare system and the need for large‐scale or long‐term clinical trials, the integration of digital therapeutics, digital biomarkers, and pharmacovigilance frameworks represents a promising and still underexplored area of research that may substantially contribute to the development of precision medicine approaches in NMDs.

## Graphical abstract

Digital innovations are revolutionizing neuromuscular disease management by integrating wearable sensors, mobile applications, and telemedicine to enable continuous monitoring and personalized care. These technologies enhance diagnosis, treatment, and drug development fostering dynamic interaction between patients and clinicians paving the way toward precision medicine and improved outcomes in neuromuscular disorders. The Graphical Abstract Image was created with Biorender.com, with publication license agreement number: TO2A5QHK4U.

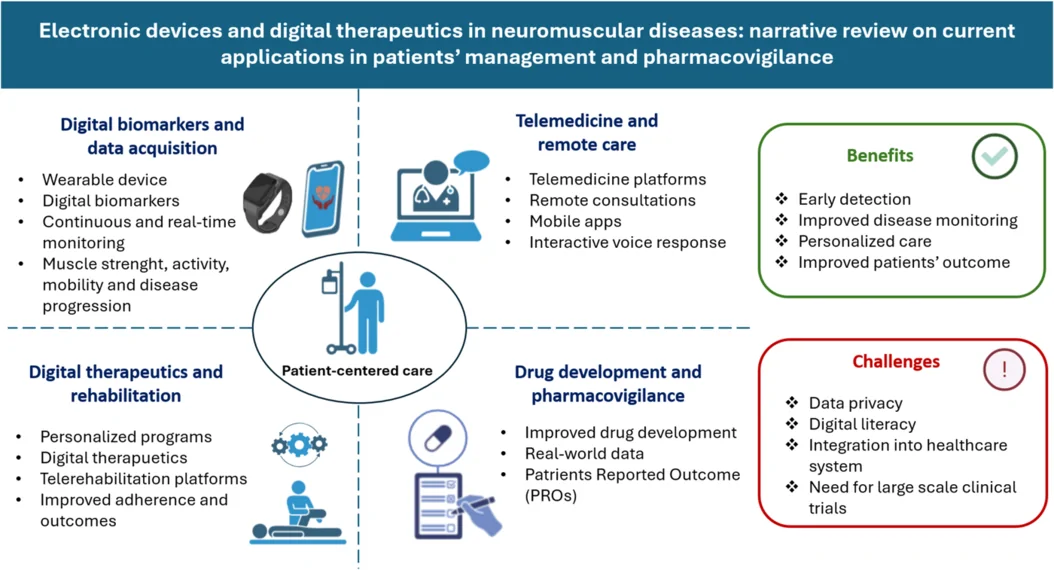

**Abbreviations**
3D: three‐dimensional
ADR: adverse drug reaction
ALS: amyotrophic lateral sclerosis
DM: dystrophia myotonica
DM1: myotonic dystrophy type 1
DMD: Duchenne muscular dystrophy
DTx: digital therapeutics
IVR: interactive voice response
NDM: non‐dystrophic myotonia
NMD: neuromuscular disease
PRO: patient‐reported outcome
SEG: sensor‐engineered glove
SMA: spinal muscular atrophy

## 1 Background

Digital health technologies, including electronic devices and digital therapeutics (DTx), have greatly improved management of diseases. The issue began with basic tools, such as devices for symptom monitoring, sensors, and software that enhanced the efficiency of healthcare systems and supported clinicians in managing patient data and care [1, 2, 3].

Wearable technology and smartphone applications allowed continuous assessment of main health parameters (e.g., heart activity, glucose levels, blood pressure, muscle activity, mobility) providing patient monitoring and personalized treatments [4, 5]. Additionally, telemedicine and remote monitoring facilitated interactions between clinicians and patients, thus reducing in‐person visits and improving treatment compliance, therapeutic efficacy, and health management [6].

These digital technologies were further implemented to act as therapeutic interventions, leading to the so‐called DTx. These digital health technologies (e.g., software, mobile applications, digital platforms, and wearable devices) were applied as therapeutic treatments to manage, treat, or prevent a number of diseases [7, 8]. DTx refer to evidence‐based therapies approved in clinical trials. Indeed, the approval of a given DTx is similar to that of a drug following preclinical research, clinical trials in randomized studies, and post‐marketing surveillance [9]. Many DTx imply a non‐pharmacological approach that targets symptoms, behaviors, attitudes, emotions, or movements associated with a variety of diseases including neurological, neuromuscular, and psychological ones [10, 11]. Thus, DTx overlap highly personalized therapies that are designed to prevent disease progression and improve quality of life [7, 12]. In parallel with their development, DTx need approval by regulatory bodies (e.g., European Medicines Agency, United States Food and Drug Administration) through specific procedures and methodologies [13, 14].

Overall, the integration of digital health technologies and DTx enables personalized patients’ monitoring, enabling tailored interventions to meet patient's specific needs [15]. Nowadays, this is important for managing chronic and progressive diseases, e.g., diabetes, cardiovascular diseases, mental health disorders, and neuromuscular diseases (NMDs) [16]. In particular, NMDs may be characterized by episodic and/or progressive muscle weakness and motor dysfunction that require real‐time monitoring and personalized interventions to manage symptoms and improve patients’ outcome [17]. Over the last decades, digital technologies have progressively emerged as valuable tools for the management of NMDs. This growing interest led to a significant increase in scientific publications exploring the clinical applications and the potential benefits of such technologies. Nevertheless, despite the increasing evidence, the available data are fragmented across different technologies and clinical applications highlighting the need for structured mapping in disease specific domains such as NMDs [18]. This narrative review aims to provide an integrated overview of the current applications of digital health technologies in NMDs as interconnected tools of a broader digital health ecosystem supporting diagnosis, monitoring, treatment, and pharmacovigilance in NMDs. This integrated view supports multiple aspects of patient care, including diagnosis, disease monitoring, rehabilitation, treatment optimization, as well as drug development and pharmacovigilance. Attention was paid to their potential involvement in precision medicine and personalized care as well as to current challenges and future perspectives.

## 2 Neuromuscular Disease

NMDs are heterogeneous disorders that affect the peripheral nervous system including motor neurons, neuromuscular junctions, and muscles. NMDs lead to progressive muscle weakness, stiffness, cramps, and pain, with variable severity depending on the pathogenesis and patient's genotype. Their clinical picture usually includes motor impairment, mobility limitations, respiratory complications, and progressive disability that result in reduced life expectancy. NMDs can be either genetically inherited or secondary to other diseases (e.g., autoimmune, inflammatory, cancer or degenerative diseases) [16, 19].

Among the genetically inherited NMDs, Duchenne muscular dystrophy (DMD) is an X‐linked disorder caused by mutations in the dystrophin gene, leading to progressive muscle degeneration, loss of ambulation, and severe cardiopulmonary complications [20, 21]. Spinal muscular atrophy (SMA) is caused by homozygous deletions or mutations in the survival motor neuron gene (*SMN1*) characterized by progressive motor neuron degeneration and muscle weakness, with a severity spectrum ranging from infantile to adult‐onset forms [22]. Myotonic dystrophy type 1 (DM1) is a multisystemic disorder caused by the expansion of cytosine‐thymine‐guanine repeats in the *DMPK* gene. It leads to muscle stiffness (myotonia) and muscle weakness but also to cognitive, cardiac, endocrine and circadian rhythm dysfunction [23]. Muscle channelopathies (e.g., myotonia congenita, sodium channel myotonia, periodic paralyses) are due to mutations in ion channels genes (chloride, sodium, calcium or potassium ones), characterized by altered muscle excitability and/or impaired excitation/contraction coupling, causing episodic muscle stiffness or paralysis, weakness, and/or congenital myopathies [24, 25, 26, 27]. The amyotrophic lateral sclerosis (ALS) is one of the most common acquired NMDs characterized by progressive degeneration of upper and lower motor neurons, resulting in muscle weakness, respiratory failure, and reduced survival [28]. Myasthenia gravis is an acquired autoimmune NMD in which the neuromuscular junction is disrupted by the immune system. Variable muscle weakness involving eyes, throat, and limbs that worsen after a period of rest is a common clinical picture. Myasthenia gravis affects young women or older men [29].

Each NMD poses specific issues and challenges due to its peculiar genotype/phenotype, difficulties in diagnosis, limited therapeutic options, and need for multidisciplinary and specialized assistance. The clinical heterogeneity and overlapping symptoms make more challenging their diagnosis [30]. To date, early diagnosis is still one of the main goals because it ensures early therapeutic intervention and prevents muscle degeneration and disease progression. Recent progress in diagnostic techniques such as advanced genetic diagnosis, muscle biopsy, and electromyography have enabled earlier diagnosis and a more accurate detection of disease symptoms [31].

Treatment strategies aim at managing symptoms, slowing disease progression, and improving quality of life. While some NMDs such as DMD and SMA can be treated with some efficacy through the gene therapy, enzyme replacement therapy, and small molecule drugs, many NMDs still have no effective treatment or have only restricted therapeutic options (e.g., mexiletine for muscle channelopathies) [32, 33, 34, 35]. Additionally, many of these drugs are expensive and not easily available, and most of them are designed as “orphan drugs” [36]. Finally, the management of NMDs often requires additional therapeutic interventions including physical therapy, rehabilitation, speech therapy, and respiratory support requiring an interdisciplinary team of clinicians (e.g., neurologists, physiatrists, respiratory therapists, geneticists) [37, 38, 39].

Based on the chronic and progressive nature of NMDs, early diagnosis, real‐time monitoring, individualized treatment, and specialized assistance are essential for ensuring the patient's care. In this context, digital innovation is currently providing increasing opportunities for diagnosis, disease management, and therapies paving the way for more effective treatments and improved outcomes of patients with NMDs.

## 3 Literature Search Strategy

This narrative review focuses on the current applications of digital health technologies in NMDs. Relevant articles were identified by interrogating PubMed using combinations of keywords including “neuromuscular diseases,” “digital health,” “digital therapeutics,” “telemedicine,” “wearable devices,” “remote monitoring,” “telerehabilitation,” and “pharmacovigilance.” Additional articles of interest were identified from the references list of selected articles. Bibliometric and systematic mapping approaches, including bibliometric network analyses, have been widely adopted to structure research landscapes in rehabilitation sciences, offering robust frameworks to identify thematic clusters, influential publications, collaborative networks, and emerging research gaps [40, 41].

## 4 Digital Health Ecosystem in NMDs

The main applications of digital innovation in NMDs are illustrated in Figure 1 and summarized in Table 1.

**Figure 1:**
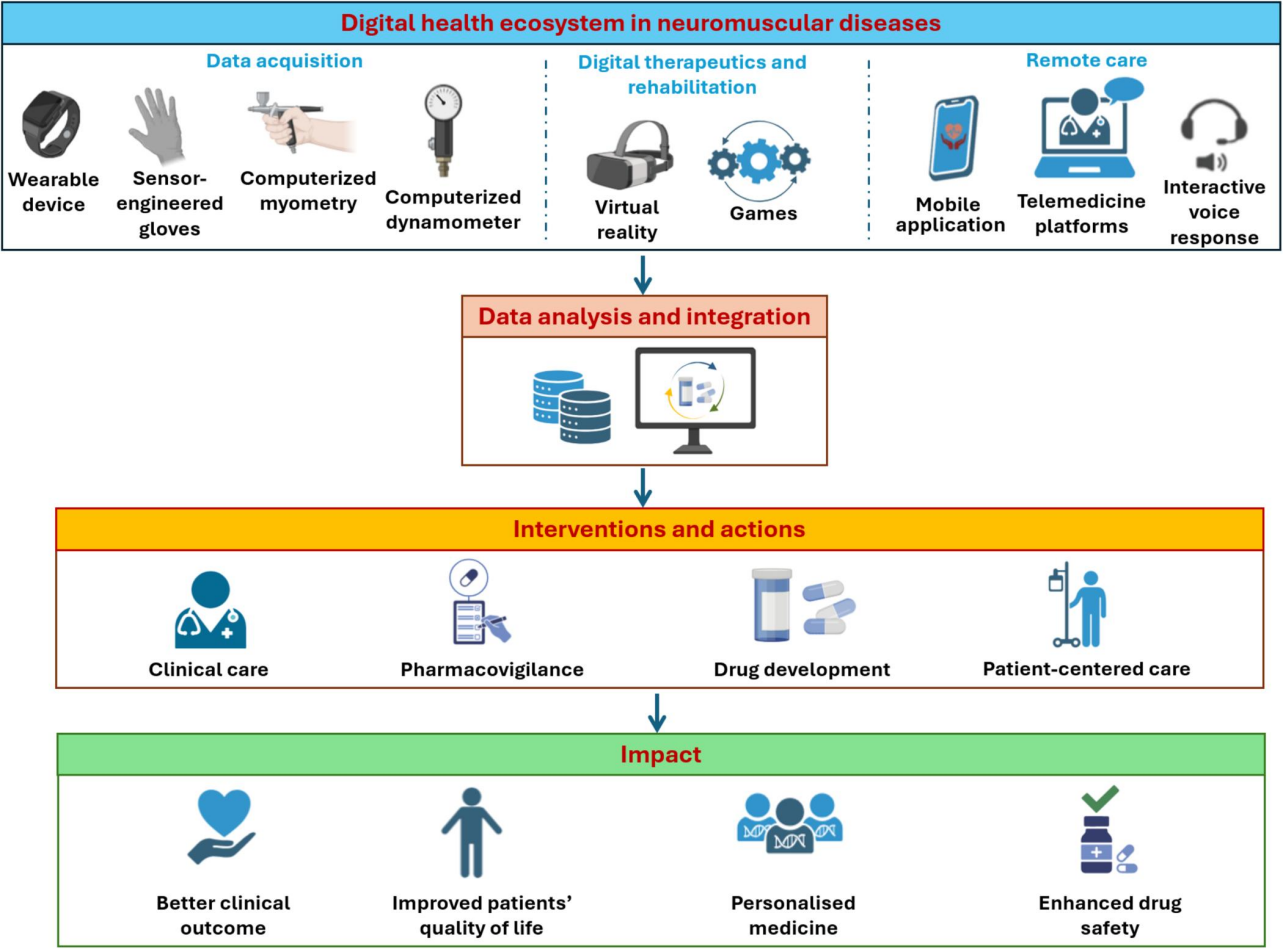
Schematic representation of the digital health ecosystem in neuromuscular diseases. Digital technologies generate patient‐derived data through symptom monitoring, digital therapeutics, and remote care, which are integrated and analyzed to support clinical interventions, pharmacological research, pharmacovigilance activities, and personalized patient management, ultimately improving clinical outcomes and quality of care. This figure was created with Biorender.com, with publication license agreement number: AJ2A5QGEDM.

**Table 1:** Digital health technologies and digital therapeutics in neuromuscular diseases: clinical applications, evidence level, and validation status.

Device/DTx	Diseases	Clinical application	Study design/evidence level	Sample size	Clinical validation status	Reference
Computerized handgrip myometry	DM1	Measures grip strength and myotonic activity through handgrip	Clinical trial	29 DM1 patients; 17 healthy subjects	Preliminary clinical validation	[42]
HandClench Relaxometer	DM, NDM	Measures muscle stiffness and relaxation in handgrip	Clinical trial	20 DM patients; 91 NDM patients	Preliminary clinical validation	[43, 44]
Sensor‐engineered gloves (SEGs)	NDM, DM	Measures grip myotonia and muscle relaxation time	Clinical trial	10 NDM patients; 10 DM patients	Preliminary and clinical validation	[45]
Actigraph (wearable accelerometer)	ALS, DMD (adults and children), DM1	Monitors mobility, posture (sitting, standing, lying), physical activity, sleep, tremor	Ongoing/approved; observational study	97 ALS patients; 60 DM1 patients	Preliminary and clinical validation	[46, 47, 48, 49]
3D accelerometer and gyroscope	DMD (children)	Posture parameters, physical activity	Pilot study	5 DMD patients	Feasibility clinical validation	[50]
EaseVRx (virtual reality DTx)	NMDs	Self‐pain management	Clinical trial	179 NMD patients	Clinical validation	[51, 52]
Telerehabilitation program (virtual reality‐based and robotic‐assisted)	DMD, Becker muscular dystrophy, ALS	Remote supervised therapy; physical activity and mobility	Clinical and experimental studies	69 DMD patients; 12 DMD and Becker disease patients; 11 ALS patients	Preliminary clinical validation	[10, 53, 54]

Abbreviations: 3D, three‐dimensional; ALS, amyotrophic lateral sclerosis; DM, myotonic dystrophy; DM1, myotonic dystrophy type 1; DMD, Duchenne muscular dystrophy; DTx, digital therapeutics; NDM, non‐dystrophic myotonia; NMD, neuromuscular disease; SEG, sensor‐engineered glove.

### 4.1 Digital Biomarkers and Data Acquisition for Diagnosis and Continuous Monitoring

Diagnosis and monitoring of NMDs are challenging because of the fluctuation of symptoms. In addition, the diagnosis is complex because it requires a combination of clinical exams, laboratory tests, electromyography, muscle biopsies, and genetic tests [55]. The evaluation of muscle function is usually performed through functional tests and subjective measures. For instance, the “myotonia test” includes manual palpation, muscle percussion, evaluation of patient's repetitive activity (e.g., grip and release fingers, close the eyes tightly, standing up and running around a chair), muscle strength evaluation using the Medical Research Council Scale, and electromyography coupled to electrical stimulation, short exercise or cooling [56]. Nevertheless, these kinds of evaluations are dependent on the clinician's experience and on the patient's ability to describe their symptoms. A major challenge may be also the episodic nature of myotonia, which may be silent during the visit. Hence, the need for standardized protocol and biomarkers has emerged to quantify symptoms and monitor the therapy efficacy. Emerging work on digital biomarkers underscores their increasing role in continuous disease monitoring and personalized care pathways [57] further supporting the use of quantitative measures of muscle function through electronic devices [58].

Electronic devices and wearable technologies provide novel opportunities for systematic and easy real‐time monitoring of patient parameters such as muscle strength, gait, posture, and respiratory function [59].

In 2007, Moxley et al. [42] examined the role of computerized handgrip myometry in assessing muscle strength and myotonia in patients with DM1. The computerized handgrip myometry consists of a digital device that records the force during contraction and the relaxation time, thus providing quantitative measure of both muscle strength and myotonic activity. Hence, a device of this type offers a more objective evaluation compared to traditional clinical methods highlighting the importance of computerized grip myometry in clinical assessment and remote management of dystrophic myotonia (DM) [42]. A similar device was investigated by Statland et al. [43] to provide quantitative handgrip myotonia as a measure of muscle dysfunction in non‐dystrophic myotonia (NDM). The handgrip myometry records a more precise, quantitative and objective measurement of muscle stiffness and relaxation time following muscle contraction, enabling the diagnosis and assessment of myotonic symptoms without any bias [43]. Also, a precise monitoring of muscle function can be obtained by the HandClench Relaxometer (National Institutes of Health Clinical Center, Bethesda, United States). This device is a simple and economical tool that provides reliable and sensitive measurements of muscle stiffness in patients with DM. It ensures remote monitoring of patients, thereby promoting home‐assistance with important implications for healthcare system and personalized care [44]. Portaro et al. [45] explored the use of sensor‐engineered gloves to measure myotonia in both patients with NDMs and DM. These gloves are equipped with sensors that measure grip myotonia and track muscle relaxation time, providing precise and objective data compared to traditional methods. Furthermore, these gloves can monitor how myotonia symptoms are influenced by external and other factors (e.g., cooling or repetitive exercises) [45].

Wearable accelerometers (e.g., Actigraph [ActiGraph LLC, Pensacola, United States]) have emerged as valuable tools for the continuous and objective assessment of motor function in patients with NMDs by monitoring physical activity, sleep, mobility, tremors, scratching, and vital signs. This digital technology is currently applied in many therapeutic areas including oncology, cardiology, psychiatry, neurology and rheumatology [60, 61]. By capturing real‐world patterns, these devices enable the generation of digital biomarkers that complement conventional clinical outcome measures and provide a more comprehensive evaluation of disease progression. Studies conducted in DMD, ALS, and DM1 have demonstrated the feasibility of using accelerometer‐derived data to monitor physical activity, movement, sleep and functional decline in both clinical and home environment [46, 47, 48, 49]. Therefore, this device may represent a valuable tool to predict disease progression and enable the accelerometer‐derived outcomes in clinical trials. In DMD patients, a pilot study assessed the accuracy of home monitoring by using a miniaturized device with a three‐dimensional accelerometer and a gyroscope positioned on the patients’ chest. This device was able to evaluate posture parameters including sitting, standing, lying and walking, thus tracking the level of physical activity in children affected by DMD. Data provided a quantification of disease impact on daily functioning and mobility enabling early and fine monitoring of symptoms and response to therapy [50].

Overall, compared with traditional clinic‐based assessments, wearable sensors offer the advantage of continuous longitudinal monitoring and may improve the sensitivity for detecting early disease‐related biomarkers enabling a more personalized therapeutic care. However, despite these promising results, the available evidence is still largely derived from feasibility, pilot and validation studies and needs further standardization in large‐scale studies. The introduction of wearable‐derived digital biomarkers into clinical practice remains limited by the lack of standardized outcome measures, well‐defined regulatory guidelines, and robust validation studies across heterogeneous NMD populations. Moreover, wearable‐derived data may be influenced by device‐specific variability, patient adherence, and differences in data acquisition protocols, potentially introducing bias and reducing comparability across studies.

### 4.2 Digital Therapeutics and Rehabilitation

Chronic muscle pain is a common feature of patients with NMDs, and its care represents a main goal of digital strategies. The management of chronic pain through digital interventions, e.g., virtual reality or gaming, has shown its significant reduction with important implications in the patients’ outcome [62, 63]. Virtual reality can modify pain perception and reduce its negative psychological effect [64]. It is a useful add‐on intervention for pain relief in patients who do not benefit from traditional analgesics due to limited efficacy or intolerable side effects [65]. For example, virtual reality‐based interventions have shown promising results in chronic pain management, improving pain perception, mood, and quality of life while supporting self‐management strategies [51, 52].

Rehabilitation and physical activity preserve mobility, enhance muscle function, and improve quality of life in NMDs. DTx and other digital health technologies, including mobile applications, wearable devices, and virtual platforms, are acquiring increasing importance as additional tools to support traditional rehabilitation approaches [66].

Recent studies have demonstrated the feasibility and potential effectiveness of telerehabilitation approaches across different neuromuscular conditions. In patients with DMD, home‐based telerehabilitation programs enabled continuity of care and personalized exercise interventions, particularly during the COVID‐19 pandemic by combining virtual consultations, remote monitoring, and digital tools [10]. Similarly, virtual reality‐based rehabilitation programs in children with DMD and Becker muscular dystrophy improved motor performance, physical activity, and quality of life by promoting engagement through personalized and interactive exercises [53]. Beyond disease‐specific programs, mobile health applications have also been developed to support home‐based aerobic exercise in patients with NMDs, providing individualized training plans, real‐time feedback, and self‐management tools that were generally perceived as user‐friendly and supportive of long‐term adherence [67]. Collectively, these findings, despite being based on feasibility and usability studies, suggest that digital rehabilitation strategies may enhance accessibility, patient engagement, and continuity of care. Other studies are currently testing robotic‐assisted rehabilitation devices to improve muscle strength and movements in patients with ALS. Although these robotic technologies are not yet approved, they support patients to recover or maintain motor function and muscle activity with encouraging outcomes in terms of improved motor coordination and general mobility [54, 68].

Overall, DTx and remote‐assisted rehabilitation strategies have shown considerable potential to improve patient engagement, accessibility to rehabilitation services, continuity of care, and outcomes in patients with NMDs but need further large‐scale randomized controlled trials and long‐term follow‐up studies to establish their clinical efficacy and introduction into routine neuromuscular care including reimbursement policies.

### 4.3 Telemedicine and Remote Care

Telemedicine uses digital communication technologies and interactive platforms to provide healthcare assistance in remote conditions. It allows patients and clinicians to interact without in‐person visits, thus reducing disparities in assistance due to physical or geographic drawbacks [69]. It has become an attractive tool for managing chronic conditions that frequently need specialist care and ongoing monitoring, including NMDs [70, 71].

A preliminary example of telemedicine was the interactive voice response (IVR) system. This allowed patients to report their symptoms and receive automated responses via telephone that resulted in a great efficacy for monitoring patients with NDM [72]. The IVR was applied in a randomized controlled trial of NDM patients treated with mexiletine, an antiarrhythmic drug used off‐label to improve myotonic symptoms. It facilitated daily monitoring by recording real‐time data on symptom severity and clinical signs of myotonia. This trial established mexiletine's efficacy in reducing the myotonic symptoms so that the drug was approved for the treatment of NDMs and designed as “orphan drug” [73, 74]. Therefore, the IVR system is a valuable tool for self‐reporting without requiring advanced technological skills. A similar telephone consultation was also applied in monitoring patients affected by myasthenia gravis and inflammatory myopathies [75]. Such a system was particularly useful in the pre‐COVID era, offering a cost‐effective and accessible tool for tracking patient conditions [76].

A turning point in telemedicine occurred during the COVID‐19 pandemic that fostered the use of digital innovations to ensure medical assistance even in the remote settings. As many patients affected by NMDs have higher risk to contract infections and develop complications, their care in remote conditions by using telemedicine and digital technologies was needed. This led to the development of advanced telemedicine platforms enabling video‐consultations, remote monitoring, wearable devices, mobile applications and virtual rehabilitation [77]. Also, these platforms ensure real‐time symptoms monitoring and response to therapy, collection of huge data, and high quality and personalized assistance even at distance.

Across different NMD populations, including patients with DMD, SMA, and ALS, remote consultations and telemonitoring programs using tablets, personal computers, grip dynamometers, and spirometers appeared feasible and were generally associated with high levels of patient and caregiver satisfaction [78, 79, 80, 81, 82]. Despite the increasing introduction of telemedicine platforms, many issues still remain to be solved: limited access to high‐speed internet, poor availability of digital devices, low grade of digital literacy especially in elderly patients. Remote consultations may not fully replace in‐person visits, in particular for complex NMDs requiring multi‐disciplinary evaluation. In addition, reimbursement policies, the interplay with existing healthcare infrastructures, and data protection requirements may further limit large‐scale implementation of telemedicine services. Nevertheless, the benefits of telemedicine (e.g., improved access to care, high‐quality and multi‐specialized assistance, reduction of hospital visits, and continuous monitoring) make it a valuable tool in the management of NMDs. Through the advent of wearable technology and artificial intelligence, the potential role of telemedicine to provide personalized assistance was further improved, offering novel and additional possibilities for patients’ management [83, 84].

Therefore, telemedicine remains a valuable component of modern NMD care, improving access to specialistic expertise, facilitating multidisciplinary management, and reducing the burden associated with frequent hospital visits. However, telemedicine should currently be considered complementary rather than substitutive to in‐person visits, in particular for complex neurological assessments. Future developments integrating wearable technologies, digital biomarkers, and artificial intelligence may further enhance remote monitoring capabilities, although additional evidence regarding long‐term clinical outcomes, cost‐effectiveness, and healthcare integration is still needed.

## 5 Drug Development and Pharmacovigilance

The digital innovations have significantly improved the landscape of drug development, from the early stages of discovery to clinical trials and post‐marketing surveillance in pharmacovigilance. Most NMDs are rare and heterogeneous diseases. They pose many challenges in preclinical and clinical pharmacological studies because of limited financial support for drug development, distance from specialized centers, difficulty to carry out clinical trials or obtain sufficient data for evaluation of drugs efficacy [85, 86].

Digital innovations have strongly improved pharmaceutical research, overcoming many of these issues. Indeed, artificial intelligence and machine learning for drug discovery and in silico drug screening significantly contribute to drug development. They are more focused and cost‐effective processes than conventional methods that accelerate identification of novel drug targets and lead compounds [87, 88]. In clinical trials and drug monitoring, wearable devices, mobile applications, and interactive platforms have significantly enhanced assessment of therapy efficacy, drug safety and potential adverse effects [89]. Wearable sensors that measure muscle strength, gait, and mobility have become invaluable tools for monitoring DMD and ALS progression. These systems provide objective, quantitative data for evaluation of the pharmaceutical therapies and sustain patient's enrollment by granting remote assistance and monitoring [46, 47, 48, 49, 50, 60, 61, 90, 91].

Digital technologies also play a key role in post‐market surveillance. Traditional methods of pharmacovigilance include spontaneous reporting by clinicians or patients with an increased probability of wrong or delay in notification of adverse drug reactions (ADRs) [92]. Instead, digital health tools allow active and real‐time monitoring of ADRs by collecting real‐life data from patients on therapy. For instance, mobile applications can foster patients to directly report side effects; wearable devices can directly track physiological responses to drugs. This approach will further support pharmacovigilance studies, leading to a prompt identification of potential ADRs [93]. Based on the continuous patients’ monitoring on disease symptoms and drug effect, digital technologies contribute to provide a patient‐centered view through the so‐called “patient‐reported outcomes” (PROs). PROs are directly reported by patients about their health condition, symptoms, and impact of treatment on their quality of life, that allow data collection independently from their interpretation by clinicians or researchers [94]. Mobile applications and other digital platforms can facilitate collection of PROs through the collection of data that reflect a more accurate analysis of the patient's response to treatment. These data will support the development of promising, safe and more personalized therapeutic approaches [95].

Digital technologies also sustain personalized pharmacological research in inherited NMDs characterized by a high genetic and phenotypic variability that leads to a variable response to therapy. Digital tools can provide useful data on genomics, clinical parameters, real‐world studies that enable the identification of genomic and/or circulating biomarkers for patients’ stratification into clusters to predict response to therapy and/or ADRs. A potential workflow for digital pharmacovigilance in NMDs may rely on the integration of multiple sources of patient‐generated health data: wearable devices, mobile applications, telemedicine platforms, and PROs can continuously collect treatment‐related information. These data may be integrated with electronic health records and analyzed through artificial intelligence and machine learning algorithms to identify safety signals, detect ADRs in real‐time, and support clinical and regulatory decisions (Figure 2). Hence, the combination of digital health data with sophisticated analytics and machine learning algorithms will contribute to the transition towards a precision medicine where drugs are developed and prescribed based on the patients’ genetics and clinical profile [96, 97, 98].

**Figure 2:**
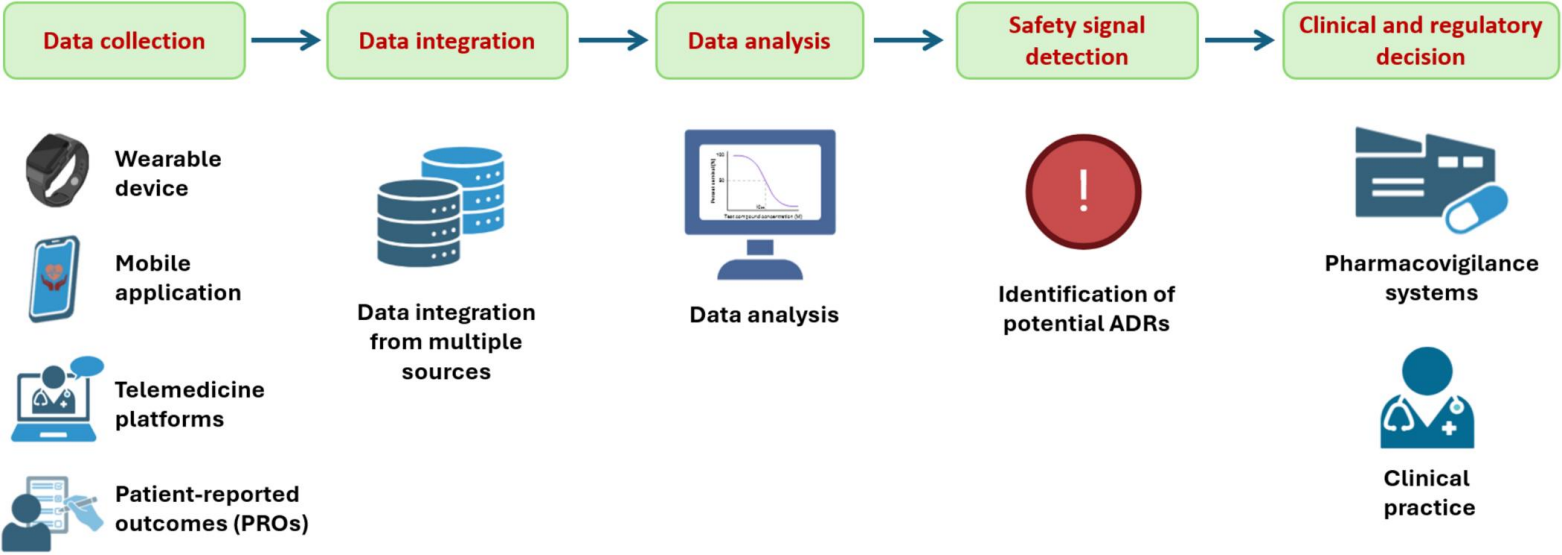
Potential workflow for digital pharmacovigilance in NMDs. This figure was created with Biorender.com, with publication license agreement number: QF2A5QH95M. ADR, adverse drug reaction; NMD, neuromuscular disease; PRO, patient‐reported outcome.

In sum, the introduction of digital technologies into pharmacological research offers several opportunities to enhance development, safety, and efficacy of drugs for NMDs. The improvement of clinical trials, real‐time data collection, and of pharmacovigilance studies will pave the way for personalized medicine and better patients’ outcomes.

## 6 Concluding Remarks, Challenges, and Future Perspectives

The introduction of digital technologies in the management of NMDs has opened new frontiers in clinical practice providing novel approaches for personalized patient care, real‐time monitoring of symptoms and disease progression, and more frequent clinician‐patient interactions. Hence, digital health technologies, including wearable devices, mobile applications, telemedicine platforms, and DTx are novel tools of healthcare systems in 4.0 medicine that led to improved outcomes, and enhanced pharmacovigilance studies. Notably, recent advances in stretchable and body‐conformable electronics have further expanded the potential of wearable devices, offering more adaptable and patient‐friendly solutions for continuous monitoring and therapeutic interventions [99]. Of note, digital technologies have also contributed to the development of non‐pharmacological therapeutic approaches, such as rehabilitation. This technological transition has enabled the development of targeted therapies for precision and personalized medicine that provide better patients’ outcomes, an improved management of symptoms and a reduction in ADR.

Nevertheless, despite the available evidence supporting the growing role of digital technologies in NMD management, the level of evidence remains heterogeneous across different applications in terms of study design, patient populations, disease subtypes, outcome measures, and digital tools evaluated, making direct comparisons across studies difficult. To date, telemedicine represents the most established application in routine clinical practice, while the use of wearable‐device, digital biomarkers and digital therapeutics is still at a preliminary stage. Accordingly, many currently available data derive from feasibility, validation, and pilot studies involving relatively small cohorts, whereas large‐scale or long‐term clinical trials remain limited. Consequently, several technologies require further validation and large‐scale studies before their implementation in routine care, pharmacovigilance programs, and drug development.

In addition, several barriers still limit the large‐scale use of digital health technologies in NMDs, including data privacy and cybersecurity, reimbursement issues, unequal access to digital technologies and differences in digital literacy as well as difficulties in integrating digital tools into existing healthcare. Furthermore, reimbursement policies and regulatory system for digital therapeutics are still evolving. All these features may represent additional sources of bias, potentially limiting the full introduction of digital technologies into clinical practice.

Many efforts are currently addressing these issues to fully accomplish the potential of digital technologies that are becoming a valuable support to the conventional management of NMDs in many fields. For clinicians, these tools may promote continuous monitoring, remote assessment, and more personalized care. For researchers, the ability to collect continuous, real‐world, and remotely acquired data offers valuable opportunities to better characterize disease progression, identify and validate digital biomarkers, and generate robust evidence‐based data through multicenter studies. From a healthcare perspective, the successful integration of digital health solutions will require appropriate regulatory frameworks, reimbursement strategies, interoperability across healthcare infrastructures, and efforts to reduce inequalities in digital access but addressing these challenges will be essential to translate technological innovation into significant clinical benefits for patients with NMDs. Therefore, future research should focus on these challenges: (1) the development and validation of standardized digital biomarkers through large multicenter and longitudinal studies, enabling their clinical relevance in both clinical practice and drug development; (2) integration of data analysis through artificial intelligence and machine learning approaches to improve disease monitoring, patient stratification, and prediction of disease progression; (3) the development of a network able to interconnect digital health platforms, electronic health data, and pharmacovigilance systems.

Overall, the integration of digital therapeutics, digital biomarkers, and pharmacovigilance frameworks represents a promising and still underexplored area of research that may substantially contribute to the development of precision medicine approaches in NMDs. The introduction of digital health technologies into neuromuscular care has the potential to improve disease monitoring, facilitate personalized interventions, strengthen pharmacovigilance activities, and expand access to specialized care providing measurable benefits for patients with NMDs.

## Author Contributions

**Ilaria Saltarella:** conceptualization, investigation, writing – original draft, writing – review and editing, data curation. **Concetta Altamura:** investigation, writing – review and editing. **Paola Laghetti:** investigation, writing – review and editing. **Giuseppina Settanni:** investigation, writing – review and editing. **Nicola Savino:** investigation, writing – review and editing. **Angelo Galiano:** investigation, writing – review and editing. **Bodvaël Fraysse:** investigation, writing – review and editing. **Angelo Vacca:** investigation, writing – review and editing. **Jean‐François Desaphy:** writing – review and editing; conceptualization.

## Ethics Statement

The authors have nothing to report.

## Consent

The authors have nothing to report.

## Conflicts of Interest

Giuseppina Settanni is employed as R&D Manager and Senior Researcher at Dyrecta Lab. Nicola Savino is employed as Executive Director and Senior Researcher at Dyrecta Lab. Angelo Galiano is employed as Chief Executive Officer and Researcher at Dyrecta Lab. The remaining authors declare no conflicts of interest.

## Data Availability

Data sharing is not applicable to this article as no datasets were generated or analyzed in the current article.
